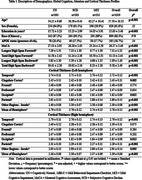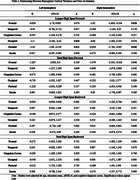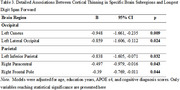# Thinner Cortex, Sharper Attention? Paradoxical Links Between Cortical Thinning and Attentional Capacity in Cognitive Aging

**DOI:** 10.1002/alz70861_108579

**Published:** 2025-12-23

**Authors:** Yi Jin Leow, Nagaendran Kandiah

**Affiliations:** ^1^ Dementia Research Centre (Singapore), Lee Kong Chian School of Medicine, Nanyang Technological University, Singapore Singapore; ^2^ Neuroscience and Mental Health Programme, Lee Kong Chian School of Medicine, Nanyang Technological University, Singapore Singapore; ^3^ Lee Kong Chian School of Medicine, Nanyang Technological University, Singapore Singapore

## Abstract

**Background:**

Cognition begins with attention. Normal aging typically involves progressive cortical thinning alongside declining attentional capacity. However, emerging evidence suggests cortical atrophy does not consistently correlate with poorer cognitive performance; paradoxically, thinner cortex in specific regions may indicate more efficient cognitive processing. We investigated associations between cortical thickness and attentional performance in a preclinical Southeast Asian aging cohort to explore these paradoxical patterns.

**Methods:**

This cross‐sectional analysis used baseline data from the Biomarkers and Cognition Study (BIOCIS), comprising 1,019 community‐dwelling older adults in Singapore (mean age=57.50±10.52years;education=15.03±3.48years;61.8% female). Participants underwent neuropsychological testing and were classified as cognitively normal(CN), subjective cognitive decline(SCD), or mild cognitive impairment(MCI). Attention was assessed using the WAIS Digit Span(DS) subtest: Longest Digit Span Forward(LDSF), Total Digit Span Forward(TDSF), Longest Digit Span Backward(LDSB), and Total Digit Span Backward(TDSB). Cortical thickness measures were extracted from T1‐weighted MRI scans (3T Siemens Prisma Fit, processed via FreeSurfer v7.2.0), based on the Desikan‐Killiany atlas (averaged into six lobar groups per hemisphere).

**Results:**

MCI participants were older, less educated, and had lower global cognition and DS performance (*p* <.001), with pronounced cortical thinning (*p* <.001) in temporal and parietal regions compared to other groups.

Thinner cortex in the left occipital (*p* =.016), left parietal (*p* =.021), right frontal (*p* =.029), and right parietal regions (*p* =.039) was associated with better performance on LDSF, but not with TDSF, LDSB, or TDSB.

Subregionally, significant inverse associations with LDSF emerged in regions linked to visual processing and attentional orientation (left cuneus, *p* =.009; left lateral occipital, *p* =.024), attention and working memory integration (left inferior parietal, *p* =.032), motor planning and attentional shifts (right paracentral lobule, *p* =.043), and top‐down attentional control (right frontal pole, *p* =.044).

**Conclusion:**

Thinner cortex in specific regions was paradoxically associated with better attention span, as measured by LDSF, an indicator of peak attentional capacity. These findings support the neural efficiency hypothesis, suggesting that cortical thinning may reflect adaptive synaptic pruning and more streamlined neural processing. Alternatively, this pattern may align with the dedifferentiation hypothesis, where reduced regional specificity compensates for age‐related declines by enabling broader, distributed recruitment. Our results caution against interpreting cortical thinning in late life as uniformly pathological; instead, certain patterns of thinning may represent adaptive or compensatory mechanisms in cognitively resilient individuals.